# Supplementary material for: Correlative SIP-FISH-Raman-SEM-NanoSIMS links identity, morphology, biochemistry, and physiology of environmental microbes
Source: ISME Commun. 2022 Jun 30;2:52. doi: 10.1038/s43705-022-00134-3 (PMC9723565; doi:10.1038/s43705-022-00134-3)
Supplement: Supplementary file 1 — Supplemental Information [file 43705_2022_134_MOESM1_ESM.pdf]

Description of Supplementary Files

File Name: Supplemental Table 1

Description: Table of the artificial mock community Raman and NanoSIMS data highlighting the output for single cells shown in Fig. 2. Because of the limited field of view possible by NanoSIMS, not every cell analyzed using Raman was also analyzed using NanoSIMS. Both deuterium (D)-labeled and unlabeled cells are shown in the table. EC, *Escherichia coli*. MA, *Methanosarcina acetivorans*.

File Name: Supplemental Table 2

Description: Table showing all Raman and NanoSIMS data used for MMB analyses. Because of the limited field of view possible by NanoSIMS in a single image, not every cell analyzed using Raman was also analyzed using NanoSIMS. G1, G3, G4 refer to FISH probes specific for MMB groups 1, 3, and 4, respectively.

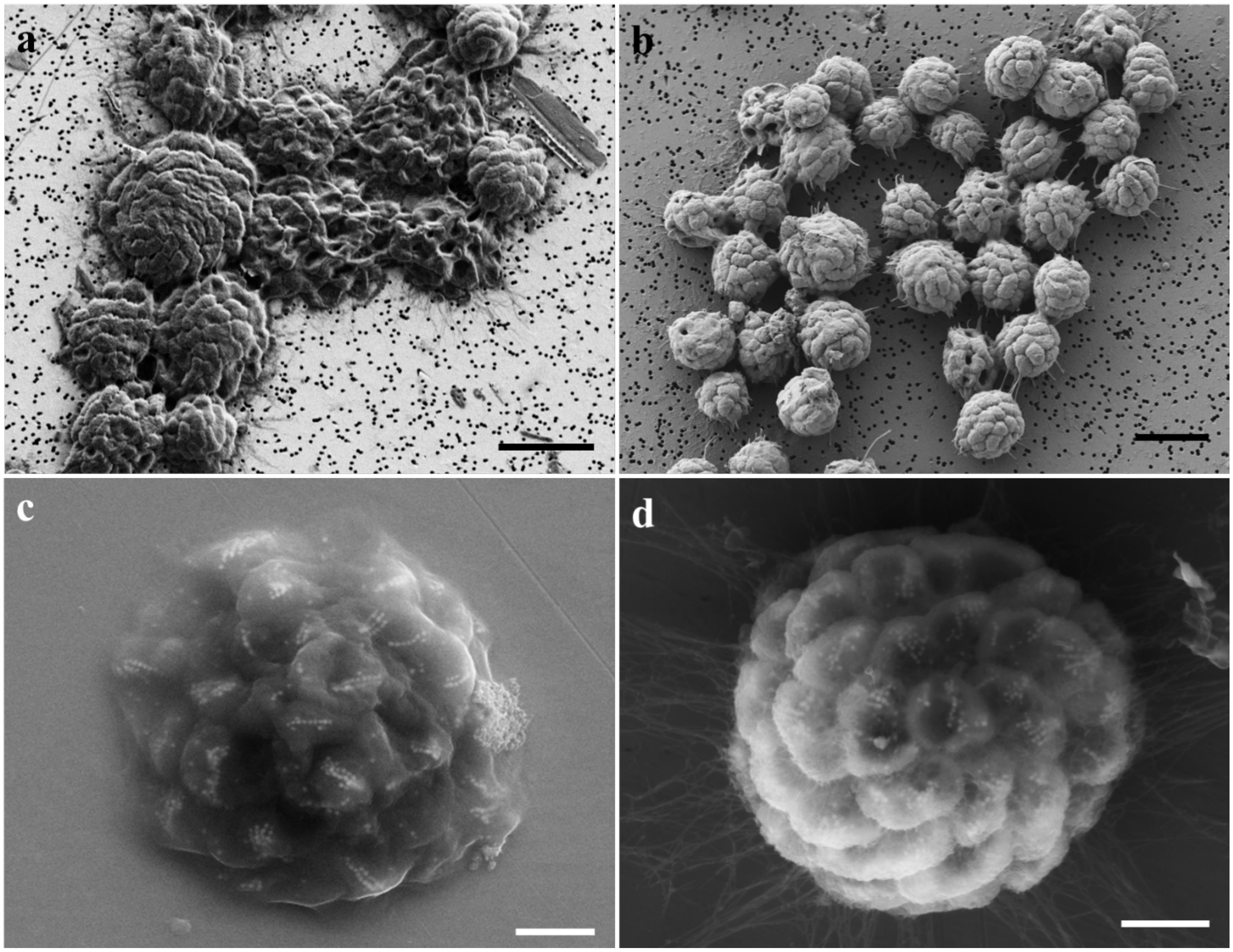

Supplemental Figure 1. SEM images showing the loss of structural integrity of MMB when FISH is performed prior to SEM. (a-b) MMB deposited on a 0.22 µm filter and imaged using SEM (a) post and (b) prior to FISH. (c-d) MMB on a stainless steel coupon imaged using back scatter electrons with the secondary electron detector (c) post and (d) prior to FISH. Scale bars in a and b equal to 5 µm, c and d equal to 1 µm.

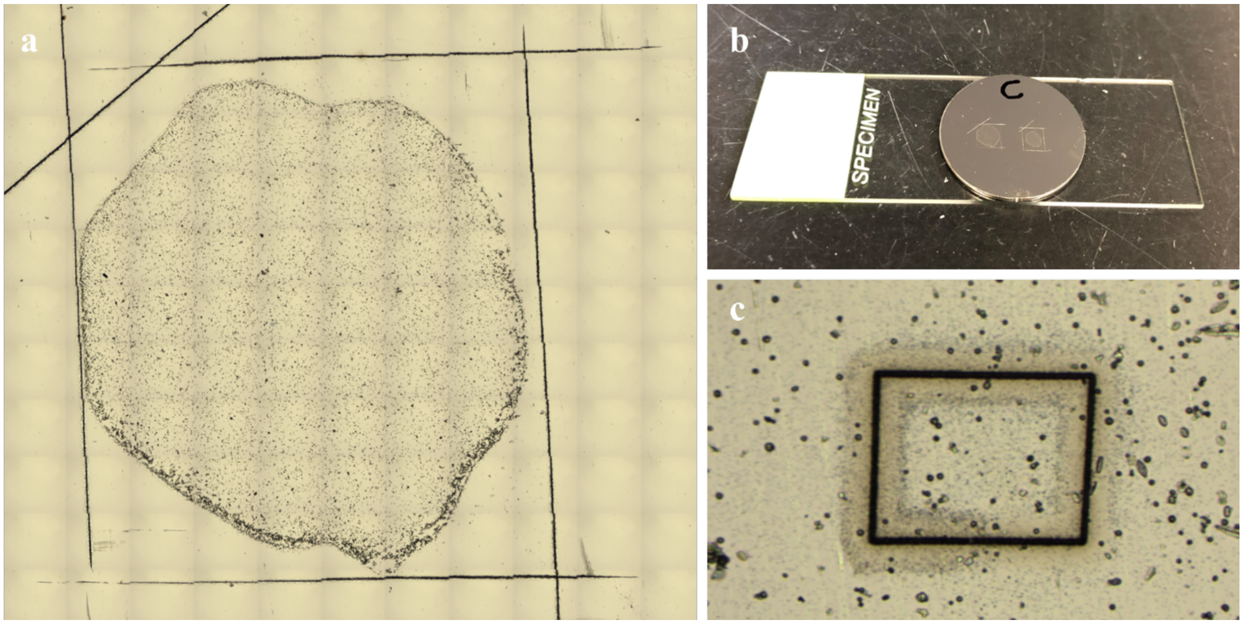

Supplemental Figure 2. Slide design and sample orientation. (a) A mosaic image showing the sample dried within an asymmetric square etched into the stainless steel coupon with a razor blade. (b) The stainless steel coupon attached to a standard 60x20 mm microscope slide. (c) An ROI that had been traced using a laser dissection microscope to assist in locating the ROI during nanoSIMS analysis.

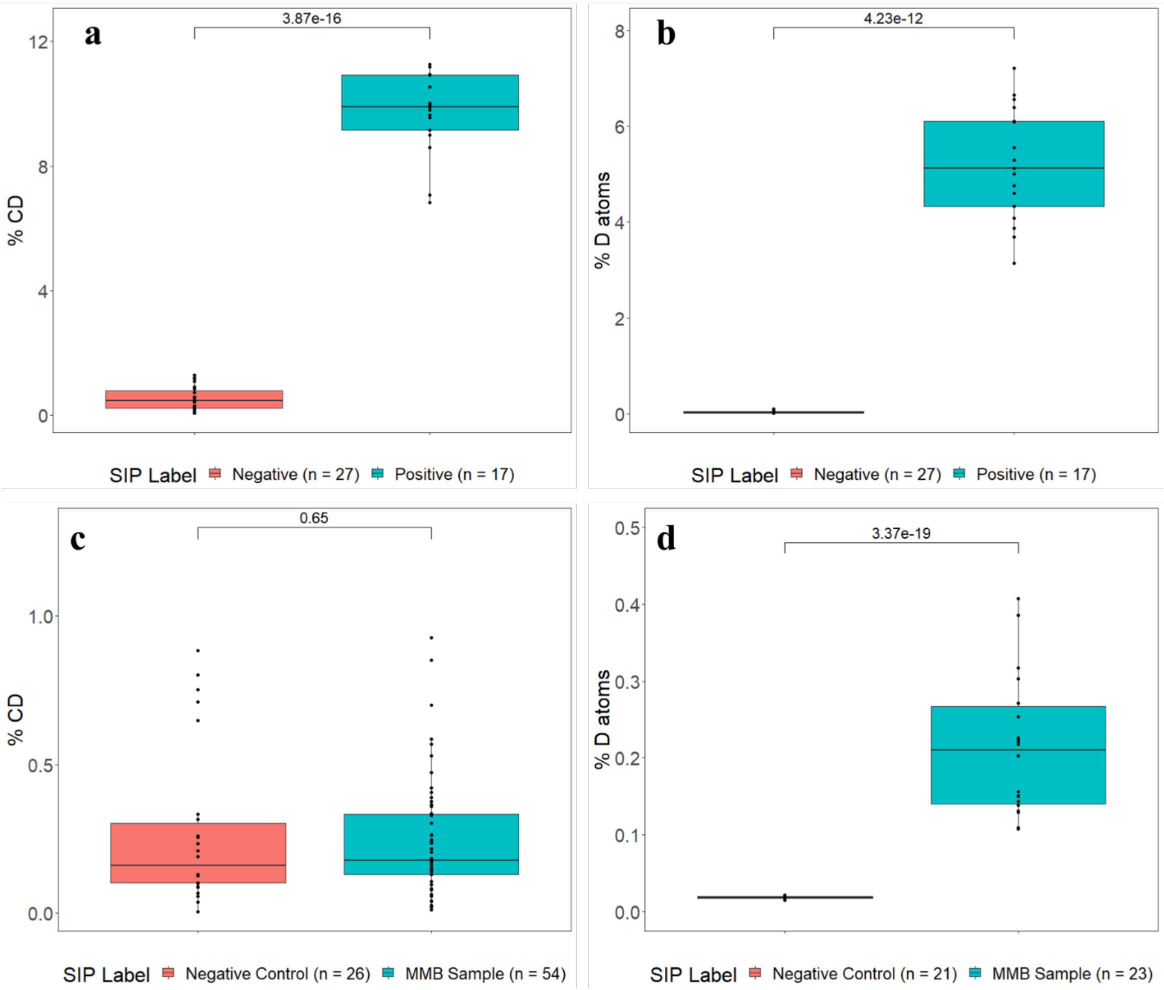

Supplemental Figure 3. Comparison of D-labeled and non-labeled cells. (a) Analysis of D-incorporation within the mock community calculated from Raman C−H (2,800−3,100 cm^{-1}) and C−D (2,040−2,300 cm^{-1}) data (p-value = 3.87×10^{-16}). (b) Comparison of the NanoSIMS m/z 2/1 (D/H) data for the same cells within the mock community shown in panel (a) (p-value = 4.23×10^{-12}). (c) Analysis of D-incorporation within the MMB calculated from Raman C−H (2,800−3,100 cm^{-1}) and C−D (2,040−2,300 cm^{-1}) data (p-value = 0.65). (d) Corresponding analysis of D incorporated into the cells using NanoSIMS; p-value = 3.37×10^{-19}) for the same MMB in panel (c). The black line represents the mean value and individual data points (*i.e.*, individual cells/MMB) are shown as black dots. Significant differences (p-value shown in plots) were determined by Students t-test.

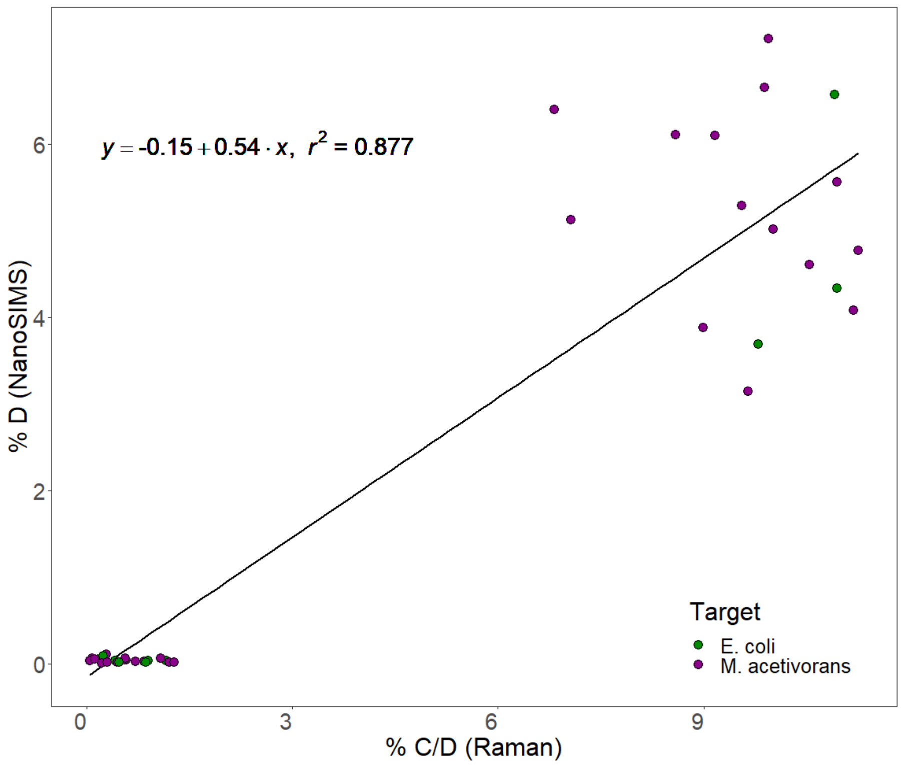

Supplemental Figure 4. Comparison of Raman and NanoSIMS atom percent of deuterium in the mock community. Both Raman and NanoSIMS were used to detect deuterium within individual cells of the mock community shown in Figure 2. Comparison of data revealed that Raman and NanoSIMS did not yield identical results on individual cells for deuterium incorporation.
